# A Case of Segmental Aplasia of the Uterus, Cervix, and Cranial Vagina in a Cat

**DOI:** 10.3389/fvets.2019.00145

**Published:** 2019-05-15

**Authors:** Samantha Souther, Nam Joo Baik, Kemba Clapp, Mike Nappier, D. Phillip Sponenberg, Julie Cecere

**Affiliations:** ^1^Mohnacky Animal Hospital, Carlsbad, CA, United States; ^2^Department of Small Animal Clinical Sciences, Virginia-Maryland College of Veterinary Medicine, Blacksburg, VA, United States; ^3^Department of Biomedical Sciences and Pathobiology, Virginia-Maryland College of Veterinary Medicine, Blacksburg, VA, United States

**Keywords:** segmental aplasia, uterus, cervix, vagina, vaginal mass, disorder of sexual development, atresia, cat

## Abstract

This case documents a rare set of congenital anomalies that resulted in an atypical cystic lesion in the cranial vagina of a queen. A discrete cystic lesion was identified in an 8 year old intact female domestic shorthair cat presenting for routine ovariohysterectomy. Morphological, radiographic, and histopathological findings were consistent with segmental aplasia of the uterus, cervix, and vagina resulting in a blind dilation of the cranial vagina. Segmental vaginal aplasia in combination with the failed canalization of the cervix resulted in a blind portion of the cranial vagina, in which normal secretions collected and became inspissated. This formed a discrete cystic structure. This case represents a novel combination and clinical presentation of segmental aplasia in the cat, involving both the uterus and cranial vagina. Ovariohysterectomy was performed for sterilization and a partial vaginectomy was performed to remove the cystic lesion in its entirety.

## Background

Normal development of the female reproductive tract requires the differentiation and canalization of tissues from different embryological origins. During female sexual differentiation, the mesoderm-derived paramesonephric ducts form the oviducts, uterus, cervix, and cranial vagina. The paramesonephric ducts develop bilaterally to form paired oviducts and uterine horns, and fuse to form the uterine body, cervix, and cranial vagina. The ectoderm-derived urogenital sinus develops into the caudal two thirds of the vagina and the vestibule. Normal vaginal development requires the fusion of the caudally-joined paramesonephric ducts and the urogenital sinus. Failure in development or fusion of the paramesonephric ducts can result in a variety of congenital anomalies including aplasia, atresia, and agenesis (1).

Failure of development and fusion of the paramesonephric ducts is rare in felines, with most cases involving segmental aplasia of the uterine horns (2). There are few reports in the existing literature describing congenital anatomical defects and abnormal development of the female reproductive tract in felines. The aim of this report is to describe a clinical case in a queen with congenital anomalies in multiple portions of the paramesonephric duct system.

## Case Report

An 8 year old intact female domestic shorthair cat was referred for evaluation of a reproductive tract anomaly identified during a routine ovariohysterectomy. The referring veterinarian discovered a large cyst-like structure of the caudal reproductive tract in close apposition to the urinary system and did not proceed with elective ovariohysterectomy. The owner reported the cat cycled regularly without noted abnormalities, but had never been bred. The cat never had abnormal vaginal discharge, pyometra, or any other significant reproductive history. The owner mentioned intermittent episodes of inappropriate urination over the past couple months, with the cat occasionally urinating outside of the litter box.

**Figure 1:**
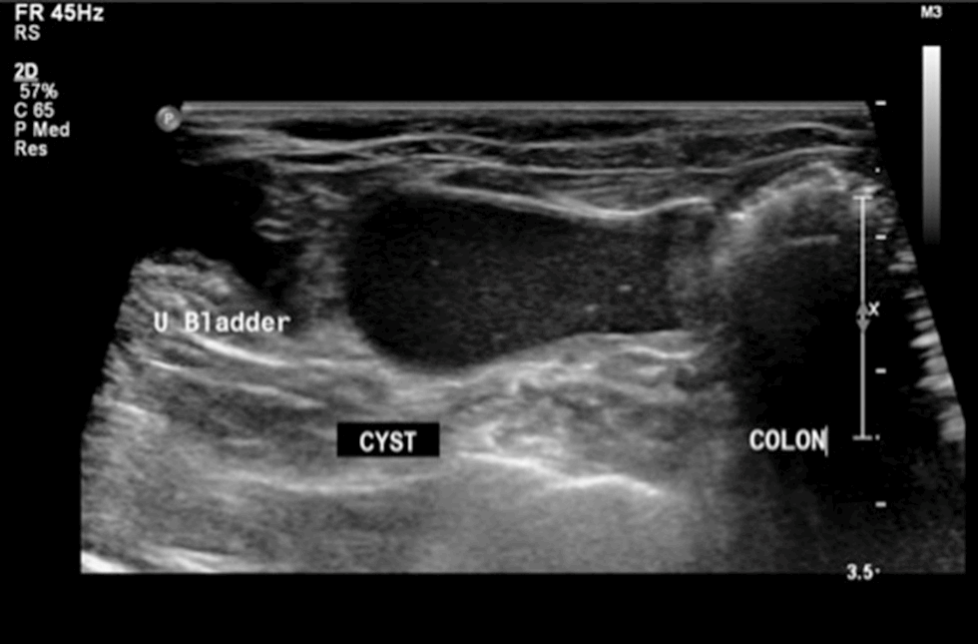
Sonographic image in transverse plane with patient in dorsal recumbency. A thin-walled cystic structure with echogenic fluid (CYST) is present in the caudal abdomen between the urinary bladder (U Bladder) and colon (COLON).

On presentation, the patient was in good condition and vital parameters were within normal limits. Physical examination revealed a non-painful abdominal distention. Evaluation of the external genitalia and mammary glands did not reveal any anatomical abnormalities. Transabdominal ultrasonography revealed a thin walled cystic structure (2.5 × 5.9 cm) containing echogenic fluid in the caudal peritoneum dorsal to the urinary bladder and ventral to the colon, with close association to the uterine horns (Figure 1). The uterine horns contained a mild to moderate amount of echogenic fluid and the uterine body could not be identified. A hypoechoic structure (0.34 cm) was found on the right ovary, consistent with a follicle. The urinary system was normal. Differentials at that time included reproductive tract cyst vs. disorder of sexual development (i.e., intersex tissue with development of paraprostatic cyst). Percutaneous ultrasound-guided aspiration of the cystic structure was extremely difficult and produced a scant amount of viscous red-tinged material with low intact cellularity, abundant necrotic debris, and anucleate keratinized squamous epithelial cells. Culture of the aspirate yielded no bacterial growth.

**Figure 2:**
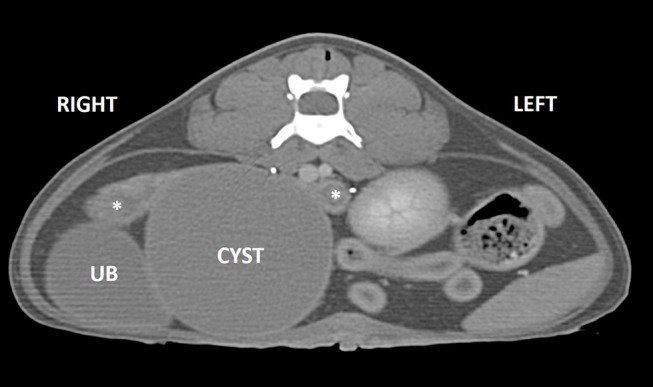
Post-contrast CT image in transverse plane. A well-marginated, peripherally enhancing cyst (CYST) is present in the region of the uterine body causing rightward displacement of the urinary bladder (UB). The mildly dilated uterine horns are seen dorsally (asterisks).

Computed tomography of the abdomen confirmed the presence of a round, well-marginated, soft-tissue attenuating, peripherally contrast-enhancing space occupying cystic lesion (4 cm H × 7.8 cm L × 4.8 cm W) in the region of the uterine body (Figure 2). Secondary effects of the space-occupying cyst included mild compression and right lateral displacement of the urinary bladder and marked ventrolateral displacement of the ureters. The kidneys were normal. The uterine horns were mildly distended with hypoattenuating fluid and converged along the dorsolateral aspect of the cyst where they then abruptly terminated along its periphery. An intramural cyst with concurrent hydrometra/mucometra was the suspected diagnosis at that time.

Blood samples were collected for preanesthetic hematological evaluation and serum biochemical analysis. Hematology revealed a mild stress leukogram (WBC 16.2 × 10^3^; Seg Neutrophils 13.68 × 10^3^) and a thrombocytosis (platelets 765 × 10^3^). Serum biochemistry showed a mild stress hyperglycemia (glucose 273 mg/dL).

**Figure 3:**
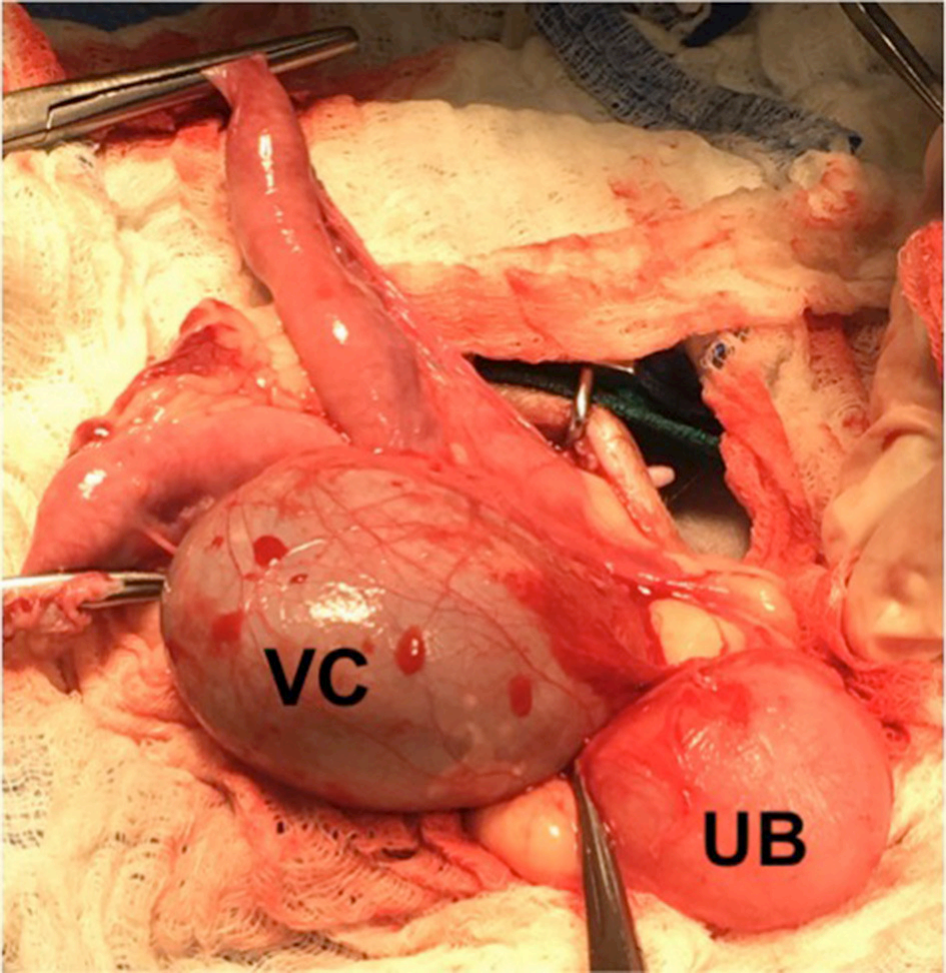
Cystic vaginal structure as viewed in surgery. The vaginal cyst (VC) encompassed the region of the cranial vagina and uterine body. The vaginal mass was closely associated with both ureters and the urethra and formed adhesions to the urinary bladder (UB).

Based on diagnostic findings the patient was admitted for an ovariohysterectomy and removal of the cystic lesion. The patient was pre-medicated and anesthetized routinely and abdominal laparotomy was performed via a ventral midline incision. The uterine horns were hypoplastic bilaterally (6 × 1 cm) and mildly distended with fluid. The ovaries were grossly normal. The ovarian pedicles were ligated and transected bilaterally. The uterus was reflected caudally revealing the discrete, round (5 × 5 cm) fluid filled structure caudal to the bifurcation of the uterus (Figure 3). The cystic structure encompassed the region of the uterine body and the cranial vagina. The cervix was not identified. Blunt dissection of the mesometrium was used to expose the cystic lesion, which had formed adhesions to the urinary bladder and was closely associated with the urethra and both ureters (Figure 3). The uterine arteries were individually ligated and a partial vaginectomy was performed in addition to ovariohysterectomy to remove the entirety of the cystic structure. The bladder was expressed verifying the patency of the urethra and ureters were traced from the bladder to the kidneys bilaterally.

**Figure 4:**
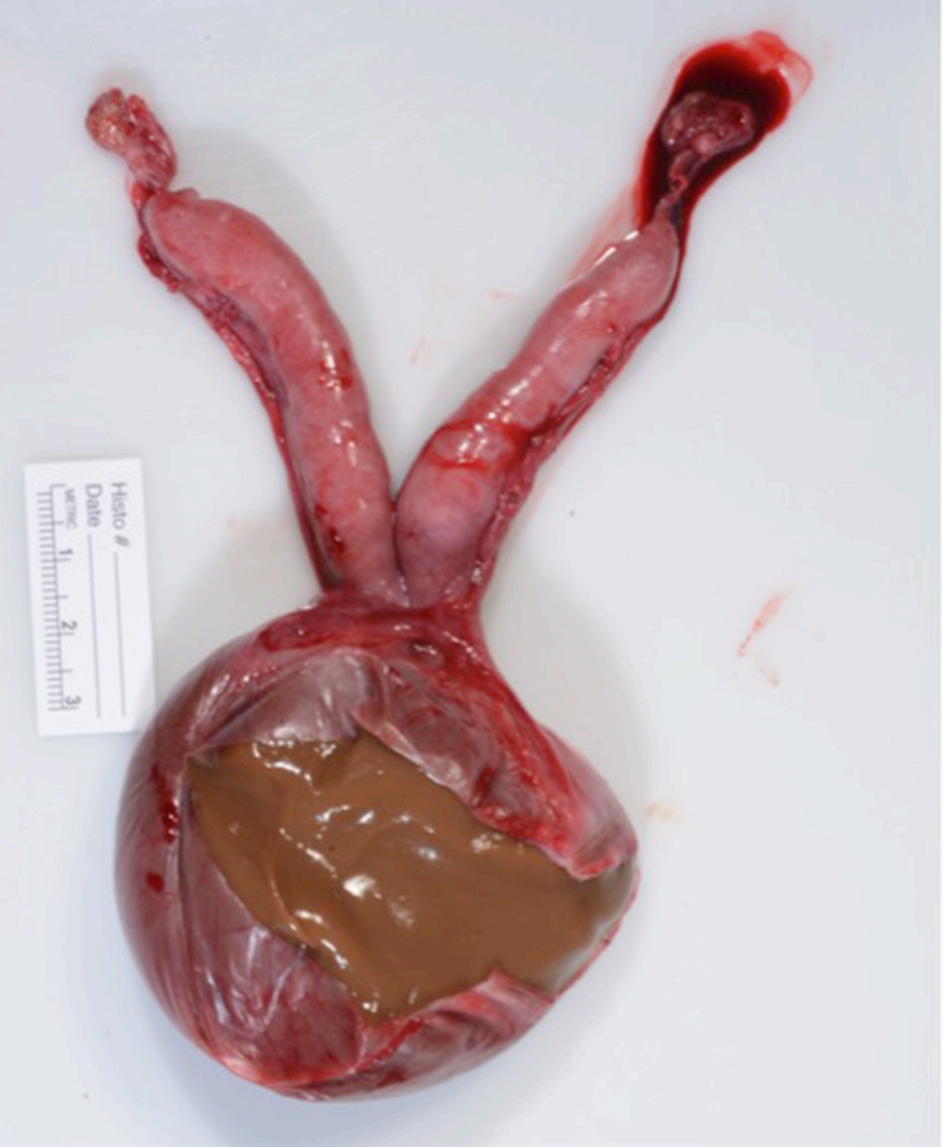
Macroscopic appearance of surgically removed reproductive tract. Ovaries are grossly normal. Uterus is hypoplasic. Cervix is not grossly visible. Vaginal cyst had been incised to reveal inspissated viscous brown material.

**Figure 5:**
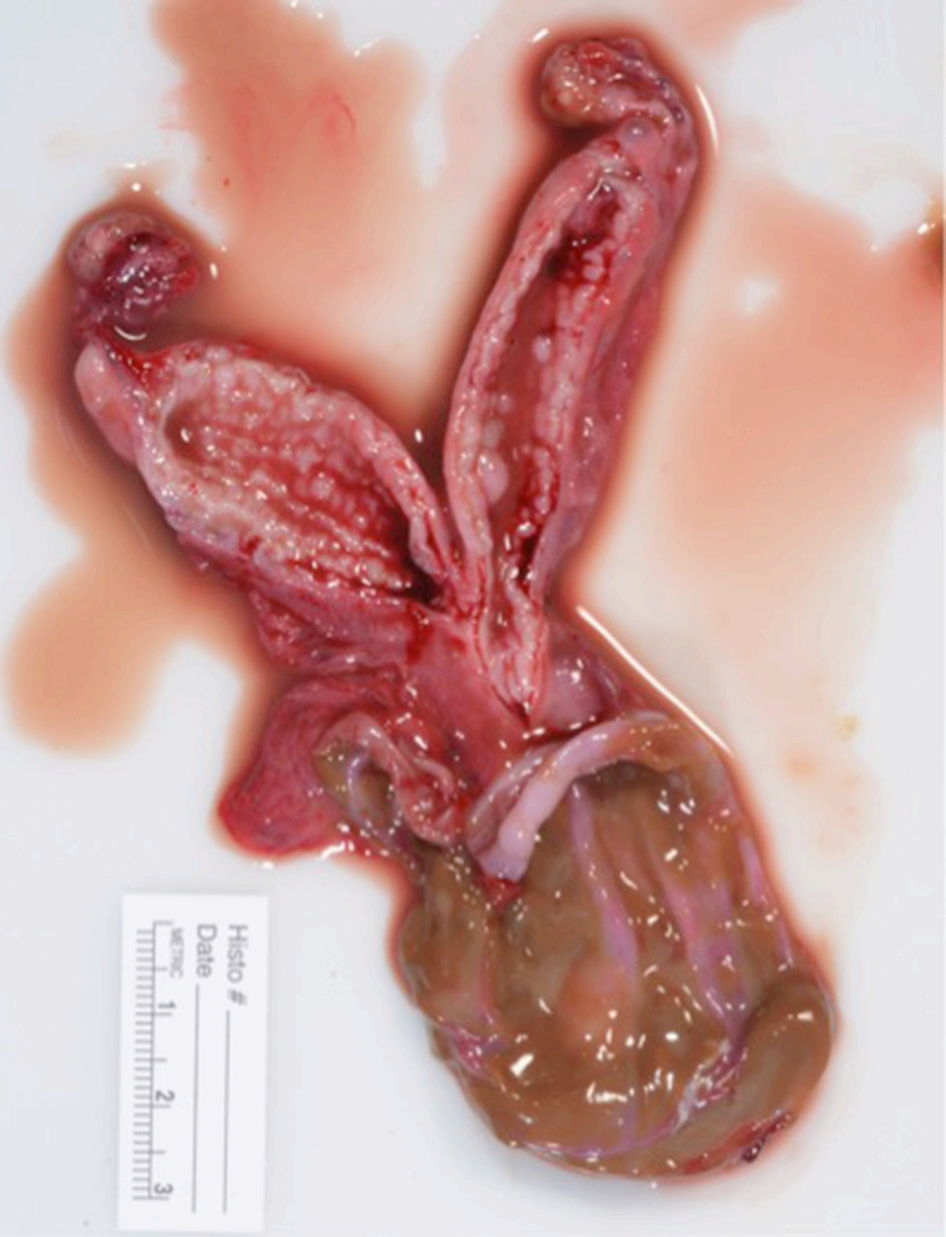
Macrosopic appearance of sagittal sections of the uterus and vaginal cyst. Dilation of uterine glands consistent with cystic endometrial hyperplasia.

**Figure 6:**
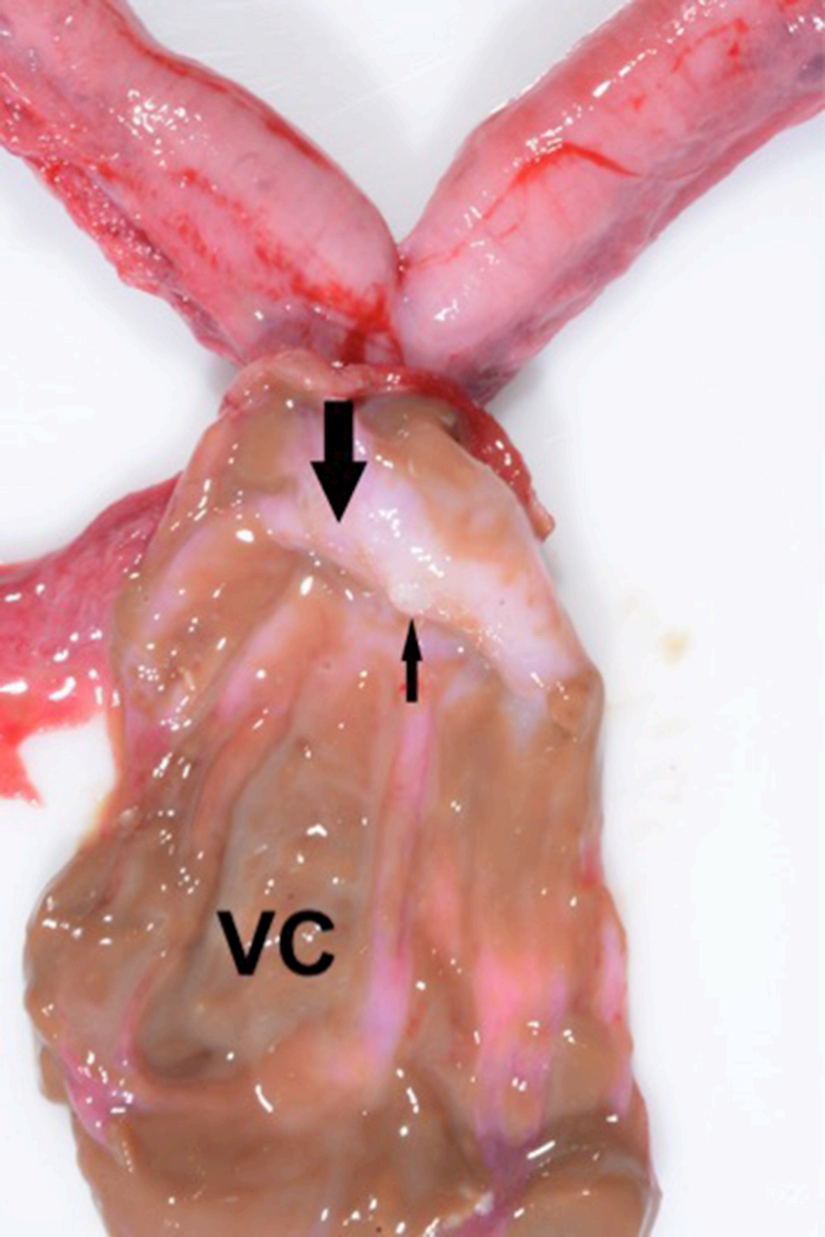
Sagittal section of the vaginal cyst (VC) revealing a fibrous septum (large arrow) in the region of the cervix. The small arrow denotes the blind end of the uterine horn. Note the absence of a patent cervical canal; no channel was evident between the uterus and the vagina.

Following surgery the excised reproductive tract was examined grossly. The cyst contained opaque, viscous brown material (Figure 4). A sagittal section of the uterus revealed diffuse cystic endometrial dilations (Figure 5). The cervical canal was not present and a fibrous septum was identified in the region of the cervix (Figure 6). The fibrous septum prevented communication between the uterus and vagina, there was no visible channel between the uterus and cranial vagina (Figure 6). The cystic lesion was located caudal to the fibrous septum, and did not communicate with the uterus grossly.

**Figure 7:**
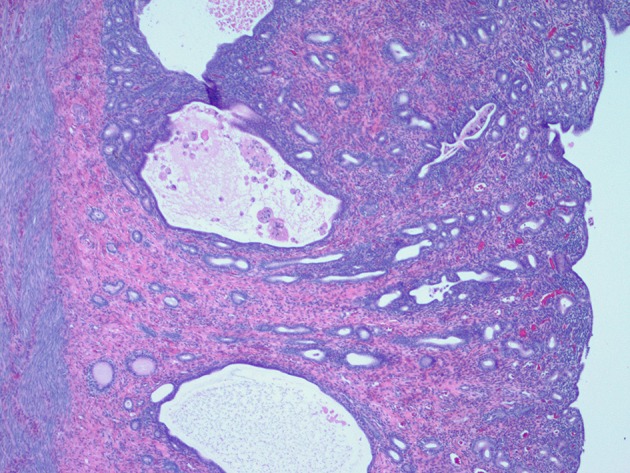
Histological appearance of the endometrium showing moderate cystic endometrial hyperplasia with scattered cystic glands with intraluminal macrophages and adjacent lymphocytic infiltration.

**Figure 8:**
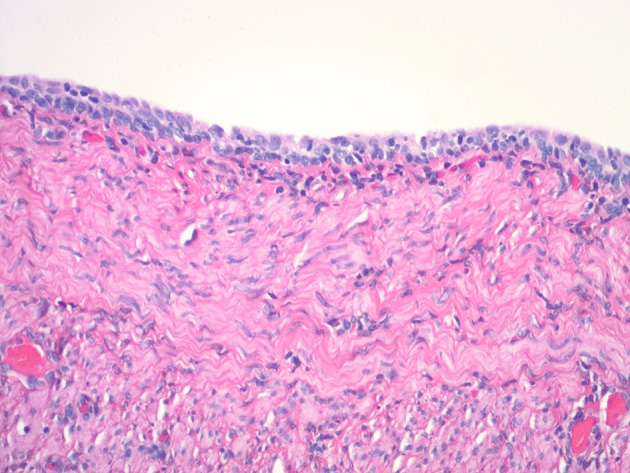
Histologic appearance of vaginal cystic structure with stratified cuboidal epithelium and wall containing smooth muscle.

The reproductive tract was submitted for histopathological evaluation. The ovaries contained several corpora lutea bilaterally. The right ovary contained a cystic structure formed of smooth muscle and lined by a single layer of cuboidal cells, probably consistent with a remnant of the male embryonic reproductive system. The mesonephric duct cyst did not appear to compromise ovarian function as the remaining ovarian stroma contained normal structures. The uterus had a moderate level of cystic endometrial hyperplasia with normal thickness and glandular development overall, scattered glands were cystic and contained small numbers of degenerate macrophages and occasional glands had tall columnar epithelium with papillary projections (Figure 7). In the endometrial interstitium were occasional foci of lymphocytes and occasional neutrophils accompanied by a very mild inflammatory reaction (Figure 7). The cystic structure of the caudal reproductive tract was formed of smooth muscle lined by a double layer of cuboidal cells, with minimal inflammatory changes (Figure 8). The contents were hyaline eosinophilic material, usually seen with proteinaceous debris. The cystic structure was determined to be a blind portion of the cranial vagina. The blind structure provided no exit for normal secretions, which accumulated and inspissated. The secretions did not contain infectious organisms.

On follow up telephone communications as recently as 3 months post-operatively, the cat was reported to be in good condition with no reproductive abnormalities. The cat still had occasional episodes of inappropriate urination, which had not changed in character following surgery and are presumed to be behavioral in nature.

## Discussion

Aplasia is the failure of a tissue or organ to develop, resulting in a rudimentary organ in an adult. Atresia is characterized by aplasia of a tubular organ resulting in stenosis or a complete absence (3). Segmental aplasia can affect multiple portions of the tubular reproductive tract due to different underlying malformations (4). Colaço et al. (4) classified the diverse causes of aplasia based on three congenital malformations: failure of the paramesonephric ducts to develop, failure of fusion of the caudal paramesonephric ducts into a single lumen, and failure of the caudal ends of the paramesonephric ducts to fuse with the urogenital sinus. In the present case the queen had aplasia secondary to all three developmental malformations, which has never been documented in one individual of this species.

Segmental aplasia of the reproductive tract is a rare finding in the cat, with cases most often involving the uterus (5). Segmental aplasia of the uterus is often identified in young animals undergoing elective ovariohysterectomy and in older queens secondary to sterility or fluid accumulation cranial to the segment of atresia (2, 5–7). McIntyre et al. (2) identified congenital abnormalities of the uterus in 0.09% of queens undergoing elective ovariohysterectomy. The developmental anomalies included unicornate uterus (complete unilateral aplasia), segmental uterine aplasia, and uterine horn hypoplasia. Additionally, ipsilateral renal agenesis was present in 29.4% of cats with congenital uterine abnormalities (8). Renal agenesis was not present in this case.

Batista-Arteaga et al. (9) described a rare case of cervical aplasia, which resulted in an exuberant mucometra in the uterus of a 14 year old queen with concurrent cystic endometrial hyperplasia. Similar to the current case cervical atresia resulted in the formation of a fibrous septum which impaired drainage of uterine fluid. In the aforementioned case it was hypothesized that estrogenic stimulation from follicular ovarian cysts contributed to the development of cystic endometrial hyperplasia and enhanced uterine secretions, resulting in the volume of fluid in the uterus. In the current case the ovaries were presumed to be functioning normally, which could account for the mild mucometra in the face of moderate cystic endometrial hyperplasia. Furthermore, while cystic endometrial hyperplasia was present in the uterus histologically, the unique combination of segmental aplasia of the cervix and vagina, sequestered the uterus from the cranial vagina, thus preventing the ascension of vaginal bacteria, and formation of a pyometra. A cystic remnant of the mesonephric duct was present in the mesovarium, and was not considered to impact ovarian function. The cat described by Batista-Arteaga et al. (9) had segmental aplasia of the cervix, which represented a failure in fusion of the caudal paramesonephric ducts as in our case, but in contrast to our cat, no other congenital abnormalities of the paramesonephric duct system were documented. Additionally, this queen had bilateral uterine hypoplasia, representing a failure of the paramesonephric ducts to develop along with vaginal segmental hypoplasia.

Vaginal segmental aplasia is an extremely rare finding in the queen. Vaginal aplasia results from the failure of the caudal ends of the paramesonephric ducts to fuse with the urogenital sinus. Failure of canalization of the cranial and caudal vagina results in vaginal atresia originating from transverse vaginal stenosis (4). This is observed as a septum between the cranial and caudal portion of the vagina, which presents similarly to an imperforate hymen, except that the transverse septum of vaginal atresia is notably thicker (4). Segmental vaginal aplasia has also been referred to as segmental vaginal stenosis and vaginal atresia (4, 10). Only one other case of segmental vaginal aplasia has been reported in the literature. Nomura et al. (10) described fluid distention of the uterus and vagina secondary to the accumulation of secretions behind a tough connective tissue border in the mid vagina. Mild cystic endometrial hyperplasia was noted in the uterus on histology. In the cat described by Nomura et al. (10) the vagina was the only structure affected by segmental aplasia and secretions accumulated cranial to the transverse vaginal septum in the uterine horns and vagina. Our case represents a unique lesion, as segmental aplasia resulted in the formation of a discrete cystic structure in the cranial portion of the vagina. Segmental vaginal aplasia in combination with the failed canalization of the cervix resulted in a blind portion of the cranial vagina which inspissated normal secretions and dilated into a large round cystic structure. In this case the blind portion of the cranial vagina was formed by segmental aplasia of the cervix and vagina, which represents a novel combination and clinical presentation of segmental aplasia in the feline.

## Concluding Remarks

This case documents a collection of rare congenital anomalies, which resulted in an atypical cystic lesion in the cranial vagina of a queen. Morphology, imaging, and histopathological findings were consistent with segmental aplasia of the uterus, cervix, and vagina resulting in a blind dilation of the cranial vagina. Cervical aplasia cranially and vaginal aplasia caudally resulted in a blind section of the cranial vagina without an exit for normal secretions. The inspissation of normal secretions in the blind portion of the cranial vagina caused discrete cystic dilation of the cranial vagina and represents a novel lesion in the queen.

## Data Availability

No datasets were generated or analyzed for this study.

## Ethics Statement

This study was carried out in accordance with the principles of the small animal department of the Virginia-Maryland College of Veterinary medicine and the patient was provided with best practice veterinary care. Written informed consent was obtained from the cat's owner for publication of this case study and all associated figures.

## Author Contributions

SS contributed to writing the manuscript and literature review. NB and KC contributed to critical revision of the manuscript as well as interpreting and describing the imaging findings. MN performed the described surgery on the patient. DS assessed the gross and histopathologic findings. JC contributed to critical revision of the manuscript, assisted with surgery on the patient, and managed the clinical case. All authors contributed to the final review.

### Conflict of Interest Statement

The authors declare that the research was conducted in the absence of any commercial or financial relationships that could be construed as a potential conflict of interest.
